# Endotoxin Tolerance Acquisition and Altered Hepatic Fatty Acid Profile in Aged Mice

**DOI:** 10.3390/biology12040530

**Published:** 2023-03-31

**Authors:** Amanda A. Wiesenthal, Thierry M. Legroux, Chris Richter, Björn H. Junker, Anne Hecksteden, Sonja M. Kessler, Jessica Hoppstädter, Alexandra K. Kiemer

**Affiliations:** 1 Pharmaceutical Biology, Department of Pharmacy, Saarland University, Campus C2.3, D-66123 Saarbrücken, Germany; 2 Marine Biology, Institute of Biological Sciences, University of Rostock, D-18059 Rostock, Germany; 3 Biosynthesis of Active Substances, Institute of Pharmacy, Martin Luther University Halle-Wittenberg, D-06120 Halle, Germany; 4 Institute of Sports and Preventive Medicine, Saarland University, D-66123 Saarbrücken, Germany; 5 Experimental Pharmacology for Natural Sciences, Institute of Pharmacy, Martin Luther University Halle-Wittenberg, D-06120 Halle, Germany

**Keywords:** LPS tolerance, hepatic fatty acids, immune response, immunosuppression, inflammation, fatty acid elongation, liver and lung tissue

## Abstract

**Simple Summary:**

Diseases that are characterized by inflammation such as sepsis, fatty liver or severe COVID-19 are more critical in the elderly population. Endotoxin tolerance is a tolerance towards pathogen components like lipopolysaccharides from bacterial cell walls. Tolerance is developed by the host to avoid strong inflammatory responses when repeatedly being in contact with these endotoxins. We hypothesized that aging is linked to a loss of this tolerance and to elevated fat content in the liver, potentially contributing to an increased risk of inflammatory diseases in the elderly. In this study, mice underwent a treatment protocol that allowed us to study the markers of tolerance in the blood and in the tissues. Based on the markers found in the blood serum and the lung tissue, the old mice showed a clear potential for endotoxin tolerance. They also showed a different fat composition in their liver compared to the young mice. Thus, we conclude that endotoxin tolerance is not necessarily affected by advanced age, but that changes in metabolic tissue homeostasis may lead to an altered immune response in old individuals. An understanding of how the immune response changes with advanced age and changed fat metabolism is important in order to understand age-related inflammatory diseases, which will help in the development of therapies for these diseases.

**Abstract:**

(1) Background: Aging is linked to an altered immune response and metabolism. Inflammatory conditions, such as sepsis, COVID-19, and steatohepatitis are more prevalent in the elderly and steatosis is linked both to severe COVID-19 and sepsis. We hypothesized that aging is linked to a loss of endotoxin tolerance, which normally protects the host from excessive inflammation, and that this is accompanied by elevated levels of hepatic lipids. (2) Methods: An in vivo lipopolysaccharide (LPS) tolerance model in young and old mice was used and the cytokine serum levels were measured by ELISA. Cytokine and toll-like receptor gene expression was determined by qPCR in the lungs and the liver; hepatic fatty acid composition was assessed by GC–MS. (3) Results: The old mice showed a distinct potential for endotoxin tolerance as suggested by the serum cytokine levels and gene expression in the lung tissue. Endotoxin tolerance was less pronounced in the livers of the aged mice. However, the fatty acid composition strongly differed in the liver tissues of the young and old mice with a distinct change in the ratio of C18 to C16 fatty acids. (4) Conclusions: Endotoxin tolerance is maintained in advanced age, but changes in the metabolic tissue homeostasis may lead to an altered immune response in old individuals.

## 1. Introduction

The SARS-CoV-2 pandemic has substantially raised awareness that the immune response changes with age: severe COVID-19 was initially linked to advanced age and hyperinflammation, which is strongly determined by the myeloid cells [1,2,3]. Sepsis, pneumonia, cardiovascular disease, diabetes mellitus, and Alzheimer’s disease also have an inflammatory basis and are more readily and severely found in the elderly [4,5,6]. With increasing age, subcutaneous fat depots decrease, and more fat is stored in the visceral depots, e.g., in the liver tissue, leading to inflammatory diseases such as non-alcoholic steatohepatitis (NASH) [4,7]. Non-alcoholic fatty liver disease (NAFLD) has been found to be associated with sepsis mortality and changes in fatty acid metabolism [8]. Furthermore, in addition to old age, NAFLD—comprising both hepatic steatosis and NASH—as well as impaired metabolic health have been associated with severe or fatal COVID-19 cases independent of the metabolic syndrome [9,10,11,12]. Both conditions, hepatic steatosis and NASH, are associated with an increased gut permeability, endotoxemia, and inflammation [13,14] and NASH is more commonly found in old patients [15].

Endotoxin tolerance represents a mechanism to avoid an exuberant innate immune response by an impaired pro-inflammatory response of myeloid cells after an initial exposure to the endotoxin lipopolysaccharide (LPS), leading to reduced levels of tumor necrosis factor (TNF), interleukin-6 (IL-6), and interleukin-1β (IL-1β) in the cells, serum, and, in accordance, also in the mRNA levels of *Tnf* and *Il1b* in the liver and lung tissue [16,17,18,19,20,21]. Endotoxin tolerance is a hallmark of the late phase of sepsis, where the macrophages and other immune cells experience a shift from a pro-inflammatory phenotype towards an anti-inflammatory phenotype. Thus, cells such as macrophages secrete less TNF and IL-1β, but more IL-10 [21,22,23]. Macrophages from aged individuals, however, show a pro-inflammatory phenotype, leading to an increased inflammatory response to an LPS stimulus [24]. One of these pro-inflammatory cytokines is IL-6 that is upregulated in serum with increasing age [25]. Additional to its role during inflammatory responses, IL-6 has also been identified as a key player in regulating lipid metabolism by stimulating lipolysis and fat oxidation in humans [26].

Considering that senior citizens are one of the most vulnerable groups in this SARS-CoV-2 pandemic as well as in other acute inflammatory diseases such as sepsis, lung and cardiovascular diseases, communicable diseases, and NASH [5,15,27], we hypothesized that old individuals have lost the potential to develop endotoxin tolerance leading to excessive inflammation and cytokine storms. We also hypothesized that this changed immune response is accompanied by an increased level of hepatic fatty acids (FA), due to a strong link between liver inflammation and hepatic fatty acid content [28].

To test the aforementioned hypotheses, we used an in vivo LPS tolerance model in young and old mice and assessed the cytokine levels in serum and the cytokine mRNA expression in the lungs and the liver as organs strongly affected by systemic inflammation.

## 2. Materials and Methods

### 2.1. In Vivo LPS Tolerance Model

**Figure 1:**
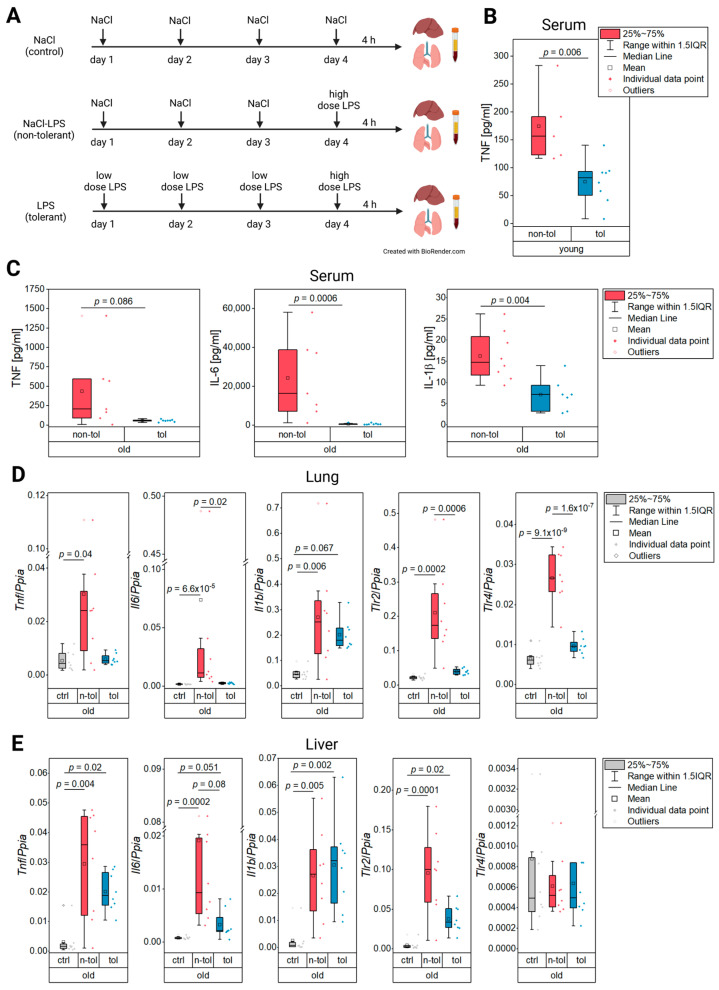
In vivo LPS tolerance model (**A**), protein levels of inflammatory cytokines in serum (**B**,**C**), and relative gene expression in lung and liver tissue (**D**,**E**). (**A**) Mice were injected (i.p.) with NaCl (0.9%), a low dose of LPS (0.5 mg/kg body weight), or a high dose of LPS (5 mg/kg body weight) on four consecutive days. Liver, lung, and serum were harvested 4 h after the final injection. (**B**) Protein levels of TNF were determined by ELISA in young non-tolerant and tolerant mice. (**C**) Protein levels of TNF, IL-6, and IL-1β were determined by ELISA in old non-tolerant and tolerant mice. (**D**) Relative expression of inflammatory markers *Tnf, Il6,* and *Il1b* as well as toll-like receptors in lung and (**E**) liver tissue.

Mice were held in IVC cages under controlled conditions of temperature, humidity, 12/12 h light/dark cycle and with food and water ad libitum. Young (10–12 weeks) and old (82–102 weeks) wild-type mice (carrying a LysM-Cre but no flox, B6.129P2-Lyz2^tm1(cre)Ifo^/J, The Jackson Laboratory) (males and females) were injected (i.p.) either with NaCl (0.9%, control) or a low dose of LPS (0.5 mg/kg body weight) on three consecutive days, followed by a high dose of LPS (5 mg/kg body weight) or NaCl on the fourth day according to their treatment group (Figure 1A). All injections were done at the same time between 8 and 9 a.m. A dose of 5 mg/kg LPS is commonly used to induce a robust but non-lethal inflammation in C57BL/6 mice [29,30]. The repeated exposure to a low dose of LPS, as seen in the tolerant group (tol), induces tolerance (Figure 1B) and is commonly used in both murine [31,32,33] and human studies [19,20,34,35,36,37,38]. Injections were made with ultrapure LPS from *Salmonella minnesota* R595 (# tlrl-smlps, Invivogen, Toulouse, France).

The livers, lungs, and serum were harvested 4 h after the final injection. The livers and lungs were immediately placed in liquid nitrogen after being harvested and stored at −80 °C until further use. Serum was also stored at −80 °C until further use. For reasons of feasibility, injections were carried out in 2 separate cohorts. This resulted in sample sizes between 7 and 8 animals (Supplementary Table S1). To ensure large enough sample sizes with mice of a certain age at a specific time point, both males and females were used (Supplementary Table S1). The local animal welfare committee (application no. 06-2016) approved all experiments.

### 2.2. Quantitative RT-PCR

RNA was isolated from small pieces of frozen liver and lung tissue with the QIAzol method (#79306, QIAGEN, Germantown, MD, USA). Preceding the precipitation of lung RNA, a final concentration of 2.5 M of ammonium acetate (#09691-250ML, Sigma, Taufkirchen, Germany) was added for a better yield. Any residual genomic DNA was removed by a DNase digestion with the DNA free kit (#AM 1906, Ambion, Waltham, MA, USA). An amount of 1 µg of the obtained RNA was reverse transcribed to cDNA using the High-Capacity cDNA Reverse Transcription Kit (#4368813, Applied Biosystems, Waltham, MA, USA) and the RNaseOUT Recombinant Ribonuclease Inhibitor (#10777019, Invitrogen, Waltham, MA, USA). qPCR measurements were carried out on CFX96 touch (BioRad, Feldkirchen, Germany) devices using HOT FIREPol EvaGreen qPCR Supermix (#08-25-00020, Solis Biodyne, Tartu, Estonia). To generate a standard curve, a plasmid dilution series of known concentrations containing the respective PCR-product was run alongside the samples. *Tlr4* constituted the only exception and was quantified using the delta Ct method. The quantities of all markers measured in the samples were normalized against *Ppia*. The gene *Ppia* was chosen among 4 other commonly used housekeeping genes on grounds of its stability throughout all age and treatment groups using the software tool geNorm [24,39]. Primer sequences are listed in Supplementary Table S2.

### 2.3. ELISA

Protein concentrations of TNF, IL-1β, and IL-6 in serum were determined by LEGEND MAX™ Mouse TNF-α ELISA Kit (#430907, Biolegend, San Diego, CA, USA), IL-1 beta Mouse ELISA Kit (#BMS6002, Invitrogen, Toulouse, France), and Interleukin-6 (mouse) ELISA Kit (#583371, Cayman Chemicals, Ann Arbor, MI, USA), respectively. Kits were used according to the manufacturers’ instructions with adjustments in serum dilution. Absorbance measurements were carried out in a GloMax Discover Microplate reader (Promega, Walldorf, Germany). For IL-6, 4 of the 7 samples in the old non-tolerant treatment group were higher than the highest concentration of the standard curve (1500 pg/mL) despite being diluted by a factor of 10.

### 2.4. Lipid Measurements

The frozen liver samples were lyophilized for 24 h with an ALPHA 2–4 LD plus lyophilizer (Christ, Osterode am Harz, Germany). The dried samples were pulverized using a ball mill (MM 400, Retsch, Germany) for 30 s at 20 Hz. An amount of 30 µL of methyl-nonadecanoat (Roth, Karlsruhe, Germany, stock solution 0.15 mg/mL in chloroform) was added to the homogenized liver samples as an internal standard and then resuspended in 600 μL methanol:chloroform:water solution (1:3:2). The mixture was shaken and incubated three times for 10 min each on ice. The mixture was then centrifuged for 11,000× *g* for 5 min at 4 °C. After phase separation, 20 μL of the lower organic phase was mixed with 60 µL TMSH (Merck, Darmstadt, Germany). All samples were analyzed by GC-TOF-MS (7890B/7200, Agilent, Santa Clara, CA, USA). One ml of the derivatized samples was injected at 250 °C in splitless mode with a helium gas flow of 1 mL/min. Chromatography was performed with a Zebron Capillary GC-Column (ZB-Semi Volatiles, length 30 m, diameter 0.25 mm, film thickness 0.25 μm, Phenomenex, Aschaffenburg, Germany). The temperature gradient was set to 60 °C for 1 min followed by a linear ramp of 10 °C/min to 320 °C and holding this temperature for 3 min. Throughout the run, the transfer line, source, and the quadrupole were set to 290 °C, 230 °C, and 150 °C, respectively. The ionization was performed with electron impact (EI) ionization and recorded in scan mode with 6.5 spectra/s.

The raw data were processed by MassHunter Qualitative Analysis software (Agilent, B.07.00) and MassHunter Quantitative Analysis software for QTOF (Agilent, B.08.00). Standard compounds were obtained from Roth (Karlsruhe, Germany) or Sigma (Taufkirchen, Germany) and used for identification and confirmation of the chromatographic peaks. Peak areas were normalized with the internal standard and dry weight of the respective compound.

### 2.5. Statistical Analysis

The statistical analysis was carried out with the software R 4.1.2 (http://www.R-project.org/, packages: ‘car’, ’FSA’, ‘tidyverse’, ‘writexl’, accessed on 2 January 2022). Data were tested for normal distribution with the Shapiro–Wilk test and for homogeneity of variance with the Fligner–Killeen test. Based on the outcome of these tests, the quantities of the ELISA results of non-tolerant and tolerant mice were either analyzed by a Student’s t-test, Welch’s t-test or Mann–Whitney U test. To compare expressed mRNA levels of *Tnf, Il6*, *Il1b*, *Tlr2*, *and Tlr4* between treatments in lung and liver tissue of old mice, either a Kruskal–Wallis followed by a post hoc test after Dunn [40] with a *p*-value adjustment after Holm [41] or a one-way analysis of variance (ANOVA) followed by a Tukey HSD post hoc test was carried out.

A two-factorial ANOVA (factor one: age, factor two: LPS pre-treatment, factor three: interaction of age and LPS pre-treatment) was used to analyze the normalized fatty acid data as well as the normalized qPCR data from genes involved in the fatty acid metabolism. It was also applied to the normalized qPCR data in lung and liver tissue of young and old mice of cytokines and toll-like receptors. Only the groups of non-tolerant and tolerant mice were included in the model. Control groups were only depicted to visualize the baseline levels. Where the two-factorial ANOVA revealed significant differences, either a one-way ANOVA with Tukey HSD post hoc or Kruskal–Wallis test with Dunn test was used to compare the levels of the various measured parameters between the groups including controls. The choice of tests was based on the fulfillment of the respective test assumptions. The significance threshold for all tests was set to *p* ≤ 0.05. Graphs were generated with OriginPro 2022b 9.9.5.167 (Academic). Individual parameters were analyzed for sex specific differences, but none were found compared to combining both sexes in the data sets.

## 3. Results

### 3.1. Serum

The TNF protein levels in blood serum from the young mice treated according to Figure 1A were significantly reduced in the tolerant compared to the non-tolerant mice (Figure 1B) indicating an LPS tolerance as was expected through the pre-treatment with LPS [19,20,36]. In the old mice, the protein blood levels of the inflammatory cytokines IL-6 and IL-1β were reduced in the mice of the tolerant treatment group. Despite the mean and median TNF levels of the tolerant group being lower than those of the non-tolerant group, a Welch’s t-test did not generate a significant difference between the treatment groups. The combined cytokine results, however, show endotoxin tolerance in the old mice (Figure 1C).

### 3.2. Lung

The cytokine levels from the serum suggested endotoxin tolerance in the old mice. Similar findings were made with *Tnf*, where the expression in the tolerant mice was just as low as it was in the control group while the non-tolerant mice had a significantly increased expression (Figure 1D). The same expression pattern was observable for *Il6* where statistical significance supported the expression decrease in the tolerant mice compared to the non-tolerant ones (Figure 1D). *Il1b* expression, on the other hand, was not altered by tolerance in the old mice as its expression was almost as high as in the mice of the non-tolerant group (Figure 1D). The pathogen recognition receptors *Tlr2* and *Tlr4* were both significantly upregulated in the non-tolerant old mice, yet showed a significant decrease in the group that received a low LPS dose pre-treatment before the final high dose compared to the animals that only received the high dose (Figure 1A,D). This significant decrease of the pathogen recognition receptors’ expression in the group of tolerant old mice again indicated that this group had a clearly suppressed immune response to a sublethal dose of LPS.

Gene expression data for the young and old mice is depicted in Supplementary Figure S1.

### 3.3. Liver

In the liver tissue, the evidence for tolerance in the old mice was not as pronounced as it was in the lung tissue as *Il1b* was not expressed in lower levels in the tolerant mice than in the non-tolerant ones (Figure 1E). Even though *Tnf* and *Il6* appeared to be decreased in the tolerant group, it was not supported by statistical evidence (Figure 1E). A similar pattern was observed for *Tlr2*, while *Tlr4* did not have any expression differences between the treatments (Figure 1E). The gene expression data for the young and old mice is depicted in Supplementary Figure S1.

**Figure 2:**
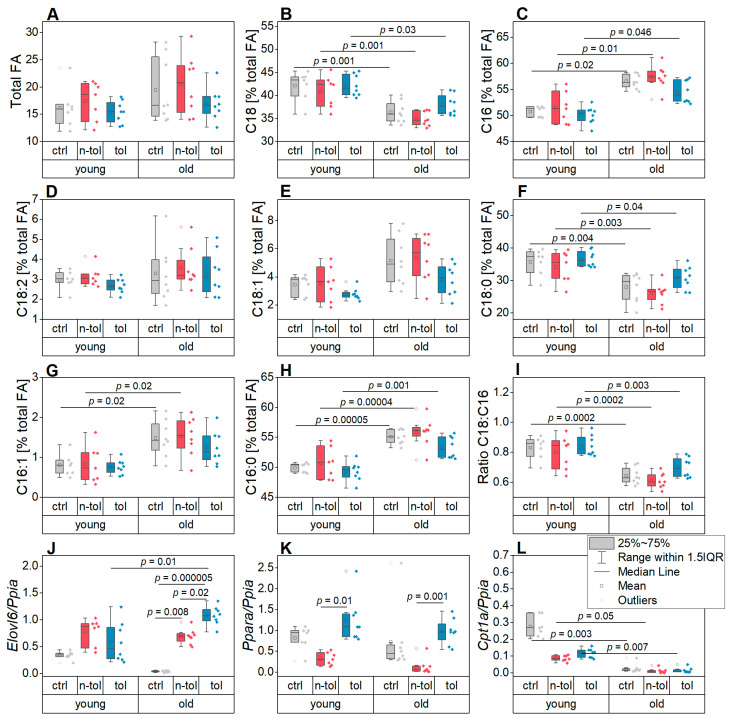
Fatty acids (FAs) and relative gene expression of relevant markers in liver tissue. Data are shown for young and old mice as well as control (ctrl), non-tolerant (n-tol), and tolerant (tol) mice in each age group. (**A**) Total amount of FAs. (**B**,**C**) Sum of all (**B**) C18 and (**C**) C16 FAs shown as percentage of the total amount of FAs, respectively. (**D**–**H**) The C18 and C16 FAs with the highest relative concentrations per mg dry tissue weight shown as percentage of total FA amount: (**D**) linoleic acid (C18:2), (**E**) oleic acid (C18:1), (**F**) stearic acid (C18:0), (**G**) palmitoleic acid (C16:1), and (**H**) palmitic acid (C16:0). (**I**) Ratio of C18/C16 FAs. (**J**–**L**) Relative expression of genes involved in lipid synthesis and lysis: (**J**) *Elovl6*, (**K**) *Ppara*, (**L**) and *Cpt1a*.

Since an altered hepatic fatty acid (FA) content typically reflects inflammatory processes in the liver [28,42], FA profiles were determined in both the young and old animals. Despite the FA content in the liver tissue not showing statistical differences between the non-tolerant and tolerant mice, according to a one-factorial ANOVA, there was an observable change between the livers of the young and old mice (Figure 2). The total amount of FAs was significantly affected by the LPS treatment, as determined by the two-factorial ANOVA, with the tolerant animals having lower levels than the non-tolerant ones as depicted in Figure 2A (Supplementary Table S3). The total FA content also appeared to be elevated in the aged mice, albeit not significantly. There were distinct changes in the FA composition between the age groups and treatment groups (Figure 2B,C,E–I, Supplementary Table S3): in the young individuals C18 FAs made up a higher percentage of the total FAs, while C16 FAs dominated in the old mice (Figure 2B,C). Accordingly, one of the most abundant C18 FAs—stearic acid (C18:0)—was decreased percentage wise in the old mice, while the two most abundant C16 FAs—palmitic acid (C16:0) and palmitoleic acid (C16:1)—were increased (Figure 2F–H). Contradictorily, oleic acid (C18:1) increased in the old animals while linoleic acid (C18:2) was not affected by age (Figure 2D,E, Supplementary Table S3). Because both the FAs, however, contribute relatively little to the total percentage of the C18 FAs compared to stearic acid, it is still significantly affected by old age leading to lower levels (Figure 2B).

The C18:C16 fatty acid ratio—frequently found to be altered in inflammatory events in the liver—was clearly higher in the young mice than the old ones, suggesting an altered fatty acid elongation activity (Figure 2J, Supplementary Table S3) [28,43,44]. This was supported by the expression of the lipogenic elongase *Elovl6* in the liver that was significantly affected by both age and the interaction of age and treatment (Figure 2J, Supplementary Table S3).

FA abundance is determined by both lipogenesis and lipolysis. We therefore assessed the gene expression of *Ppara* and *Cpt1a*, both of which are involved in FA catabolism (Figure 2K,L). *Ppara* had distinctly higher expression levels in the tolerant mice compared to the non-tolerant mice and was affected by age according to the two-factorial ANOVA (Supplementary Table S3). *Cpt1a* was also differentially expressed in the young mice compared to the old ones (Supplementary Table S3), the latter showing a higher expression (Figure 2L). The expression patterns of these FA catabolism markers suggest that they contributed to the reduced total amount of FAs in the younger age group as well as in the tolerant mice.

## 4. Discussion

Advanced age is often accompanied by impaired physical and mental health as well as an increased mortality from communicable infectious diseases [45,46]. A key player causing age-associated degenerative diseases and increased severity is inflammation, both acute and chronically low-grade [45,46,47]. In fact, severe COVID-19 was initially linked to advanced age and hyperinflammation, which is strongly determined by myeloid cells [1,2,3]. The hypothesis of an overexuberant immune response to SARS-CoV-2 as a main cause of death, however, has been challenged by studies determining immunosuppression as a critical factor in severe COVID-19 cases [48,49]. Moreover, endotoxin tolerance hallmarks are induced by SARS-CoV-2 proteins and are found in COVID-19 patients [48,49,50]. Along with the SARS-CoV-2 pandemic came a stream of research identifying the virus and its effects. Specifically, factors and causes justifying the variety in severity started drawing attention. Advanced age, obesity, type 2 diabetes, smoking, hepatic steatosis, steatohepatitis, and cardiovascular disease are all independently considered risk factors for a severe outcome of COVID-19. These pathologic conditions are often associated with an increased circulation of bacterial products such as LPS [9,10,50]. The increased circulation of endotoxins can lead to an endotoxin tolerance causing an impaired immune response and thus ineffective clearing of pathogens [50]. Endotoxemia and inflammation due to an increased gut permeability are also symptoms of hepatic steatosis and NASH [13,14]. Since these diseases are characterized by an increased level of lipids in the liver—NASH is more commonly found in old patients [15] and a strong link between liver inflammation and hepatic fatty acid content has been shown [28,42]—we analyzed inflammatory markers on a protein and mRNA level as well as the hepatic fatty acid (FA) composition.

In this study the potential of the old mice to develop an endotoxin tolerance was investigated as well as the fatty acid composition of livers from the young and old mice. In accordance with previous findings [20,36], an LPS pre-exposure decreased the serum levels of the inflammatory cytokine TNF in the young mice demonstrating an acquired endotoxin tolerance. The old mice also showed this endotoxin tolerance by a suppressed secretion of the pro-inflammatory proteins IL-6, and IL-1β in the tolerant mice with a similar tendency for TNF. This observation is in accordance with Kohman et al. [51], who found protein levels of TNF, IL-6, and IL-1β to be increased in the spleen of young (4 months) and middle-aged (12 months) male BALB/c mice after an acute LPS injection, while the levels were reduced after a repeated LPS administration. An expected higher secretion of serum TNF in the non-tolerant old mice compared to the young ones after receiving their single high LPS dose [24] was met for most data points (young: 174.1 ± 30.2 (sem), old: 434.2 ± 183.8 (sem)).

The acquired endotoxin tolerance in the old mice was not only supported by the serum protein levels of pro-inflammatory markers, but also in the expression of these markers in the lung and liver tissue as previously reported for young mice [19,20,36], albeit the effect was not as pronounced in the liver as it was in the lung tissue. While the endotoxin tolerance was observable in the *Tnf* and *Il6* expression in the lung of the old individuals, it was not statistically supported in the liver. Despite the pro-inflammatory *Il1b* not supporting an endotoxin tolerance, the expression of *Tlr2* in both tissue types and *Tlr4* in the lung tissue strongly did. Similar to Sun et al. (2012), who reported that the ability of LPS to induce TLR2 and TLR4 was reduced in peritoneal macrophages after LPS restimulation, we also observed the repeated LPS treatment affecting *Tlr2* and *Tlr4* expression in the lung tissue [52]. 

The results failing to support an endotoxin tolerance for each inflammatory marker in each tissue at the exact time point the samples were taken was to be expected, as each parameter has a different kinetic and metabolism. Thus, as the lung and the liver tissue metabolize substances very differently and are, therefore, differently affected by them, it was not surprising that we saw discrepancies in the gene expressions between these tissue types. 

Overall, the protein and mRNA levels indicated the general potential for endotoxin tolerance in the old mice, yet not to the same degree in each marker and tissue. 

A change between the age groups in the overall accumulation of FAs in the liver tissue as well as in the ratio of C18 to C16 FAs was observed. This not only indicated a fundamental change in the fatty acid composition of the liver, but by extension also a changed fluidity of the cell membranes and energy metabolism [53,54]. The increased level of palmitic acid in old mice may be a key parameter causing inflammation in aged individuals, since palmitic acid has been shown to polarize monocytes to the inflammatory M1 macrophage phenotype in healthy midlife human males [55]. The two-way ANOVA in our study not only identified age, but also treatment, to affect the total FA level. Compared to non-tolerant mice, tolerant individuals showed a reduced FA level. This may be explained by the declining IL-6 levels in endotoxin tolerance, since IL-6 is involved in regulating lipid metabolism by stimulating lipolysis and fat oxidation in humans [26]. Higher levels of IL-6, as observed in aged humans [25], may also contribute to the altered FA profile in the livers of aged mice [56].

In accordance with previous studies, old mice showed a relative increase in oleic acid resulting in a lower ratio of the unsaturated stearic acid to the monosaturated oleic acid, as observed in patients with hepatocellular cancer or NASH [28,57,58].

Altered C18:C16 ratios and the expression of the fatty acid elongase ELOVL6 have been reported to contribute to NASH [28,43,44]. Depending on the mouse model, either elevated [28] or decreased [59] ratios have been observed, while human data indicate that reduced C18:C16 are found in steatohepatitis [60]. Interestingly, it has been shown that an age-associated upregulation of circulating C16:0 can promote pro-inflammatory monocyte polarization. However, this potential link between hepatic FA metabolism and age-dependent macrophage polarization requires further investigation [55].

Overall, we found that old mice showed a clear potential for endotoxin tolerance in terms of systemic cytokine levels as well as cytokine expression in the lung and the liver. The higher total content of hepatic fatty acids and the changed ratio of C18 to C16, observed in aged mice, may modulate immune responses in this tissue.

## 5. Conclusions

We conclude that the acquisition of endotoxin tolerance is not decreased in old age and thus an altered immune response is not necessarily linked to an altered cytokine expression, but to a modified metabolism including a changed hepatic fatty acid composition. Further studies are needed to reveal the underlying mechanisms in endotoxin tolerance in old mice.

## Data Availability

Data available upon request.

## Supplementary Materials

The following supporting information can be downloaded at: https://www.mdpi.com/article/10.3390/biology12040530/s1, Table S1: Sample sizes of sample types, age groups, and LPS treatments; Table S2: Real-Time PCR primers and conditions; Table S3: Analyses of hepatic fatty acid abundance and gene expression with a two-factorial ANOVA. Figure S1: Relative gene expression in lung (A–E) and liver (F–J) tissue. Data are shown for young and old mice as well as control (ctrl), non-tolerant (n-tol), and tolerant (tol) mice in each age group. Note: Results of two-factorial ANOVA in Supplementary Table S3.
