# Supplementary material for: Endotoxin Tolerance Acquisition and Altered Hepatic Fatty Acid Profile in Aged Mice
Source: Biology (Basel). 2023 Mar 31;12(4):530. doi: 10.3390/biology12040530 (PMC10135800; doi:10.3390/biology12040530)
Supplement: Supplementary file 1 [file biology-12-00530-s001.zip › biology-2213886-supplementary.pdf]

biology

Supplementary Material to

Endotoxin tolerance acquisition and altered hepatic fatty acid profile in aged mice

Amanda A. Wiesenthal [1,2], Thierry M. Legroux [1], Chris Richter [3], Björn H. Junker [3], Anne Hecksteden [4], Sonja M. Kessler [5], Jessica Hoppstädter [1] and Alexandra K. Kiemer [1,*]

[1] Pharmaceutical Biology, Department of Pharmacy, Saarland University, Campus C2.3, D-66123 Saar-brücken, Germany
[2] Marine Biology, Institute of Biological Sciences, University of Rostock, D-18059 Rostock, Germany
[3] Biosynthesis of Active Substances, Institute of Pharmacy, Martin Luther University Halle-Wittenberg, D-06120 Halle, Germany
[4] Institute of Sports and Preventive Medicine, Saarland University, D-66123 Saarbrücken, Germany
[5] Experimental Pharmacology for Natural Sciences, Institute of Pharmacy, Martin Luther University Halle-Wittenberg, D-06120 Halle, Germany
* Correspondence: pharm.bio.kiemer@mx.uni-saarland.de

1. Supplementary Tables

Table S1. Sample sizes of sample types, age groups, and LPS treatments. Numbers in brackets represent the number of female mice within the sample size.

	young			old		
	control	non-tolerant	tolerant	control	non-tolerant	tolerant
lung	7 (4)	7 (4)	8 (4)	8 (5)	8 (5)	8 (4)
liver	7 (4)	7 (4)	8 (4)	8 (5)	8 (5)	8 (4)
serum TNF	NA	5 (3)	8 (4)	NA	7 (5)	8 (4)
serum IL-1β	NA	NA	NA	NA	8 (5)	7 (3)
serum IL-6	NA	NA	NA	NA	7 (5)	8 (4)

Table S2. Real-Time PCR primers and conditions.

**Primer**	**Forward Primer Sequence (5'→3')**	**Reverse Primer Sequence (5'→3')**	**NCBI Accession number**	**µl primer [10 µM/ 20 µl reaction]**	**Annealing Temp-erature [°C]**	**qPCR program**
Cpt1a	CTCAGTGGGAGCGACTCTTCA	GGCCTCTGTGGTACACGACAA	NM_013495.2	0.5	60	• 95°C for 15 min<br>40 cycles:<br>• 95°C for 15 sec<br>• Annealing [°C] for 20 sec<br>• 72°C for 20 sec<br>• 65°C for 5 sec
Il10	GCCCAGAAATCAAGGAGCAT	GAAATCGATGACAGCGCCT	NM_010548.2	0.5	60	
Il1b	CCAAAAGATGAAGGGCTGCTT	GGAAGGTCCACGGGAAAGAC	NM_008361.3	0.5	60	
Il6	AAGAAATGATGGATGCTACCAAACTG	GTACTCCAGAAGACCAGAGGAAATT	NM_031168.2	0.4	60	
Ppara	CCTTCCCTGTGAACTGACG	CCACAGAGCGCTAAGCTGT	NM_001113418.1	0.5	60	
Ppia	GCGTCTCCTTCGAGCTGTTT	CACCCTGGCACATGAATCCT	NM_008907.1	0.5	60	
Tlr2	CACTGCCCGTAGATGAAGTC	TACCTCCGACAGTTCCAAGA	NM_011905.3	0.5	60	
Tlr4	TCCCTGCATAGAGGTAGTTCC	TCAAGGGGTTGAAGCTCAGA	NM_021297.3	0.5	60	
Tnf	CCATTCCTGAGTTCTGCAAAGG	AGGTAGGAAGGCCTGAGATCTTATC	NM_013693.2	0.5	60	

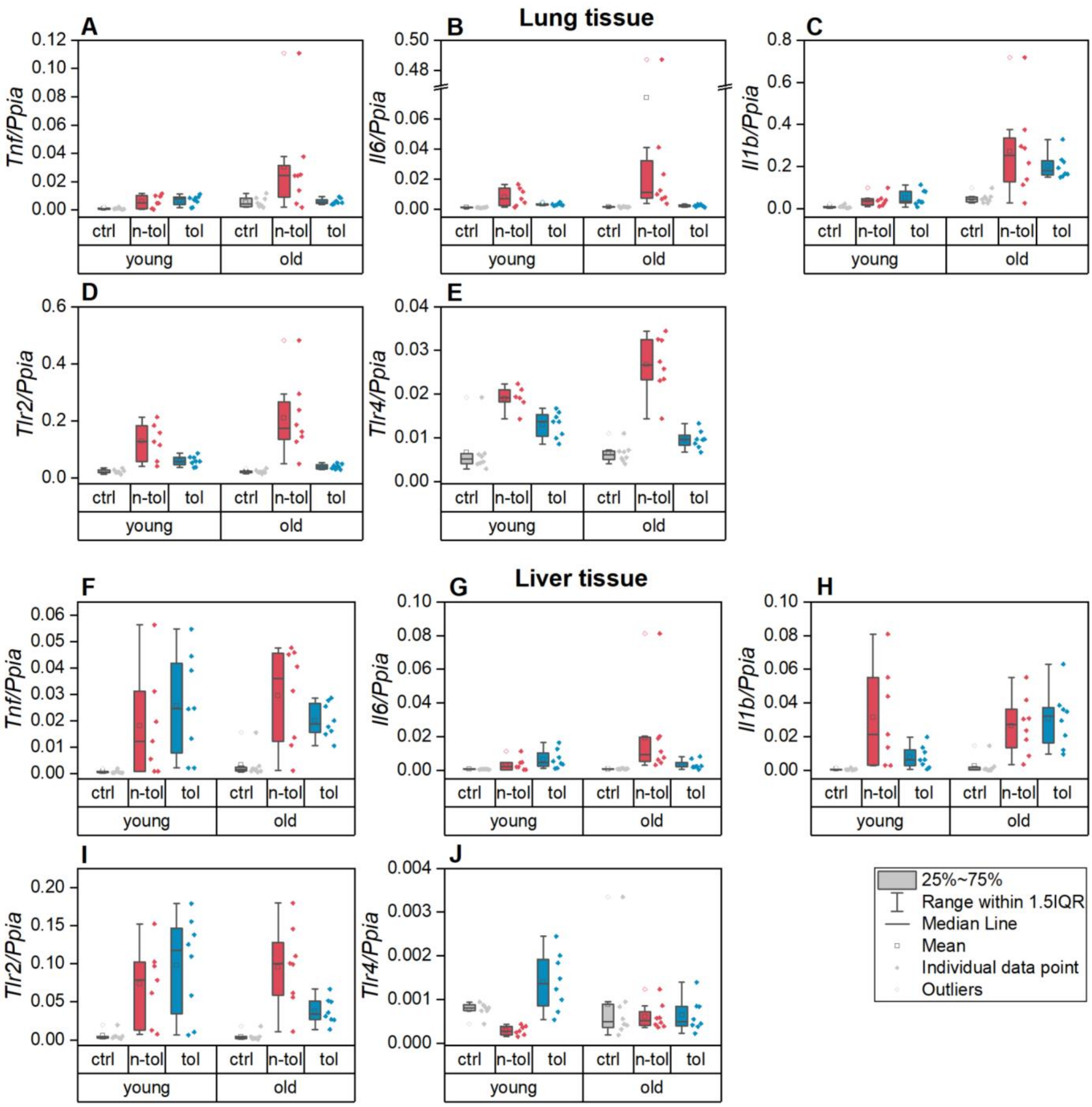

Figure S1: Relative gene expression in lung (A-E) and liver (F-J) tissue. Data are shown for young and old mice as well as control (ctrl), non-tolerant (n-tol), and tolerant (tol) mice in each age group. Note: Results of two-factorial ANOVA in Suppl. Table. S3.

Table S3. Analyses of hepatic fatty acid abundance and gene expression with a two-factorial ANOVA. Significance codes: '***' $p < 0.001$, '**' $p < 0.01$, '*' $p < 0.05$, '.' $p < 0.1$, ' ' $p < 1$. For cases where $0.05 < p < 0.1$, the exact p-value was added in brackets.

**Gene expression in lung tissue**	Age	Treatment	Age* Treatment
Tnf		**	
Il6			
Il1b	***	. (0.0658)	
Tlr2		***	
Tlr4	***	***	***
**Gene expression in liver tissue**			
Tnf			
Il6			. (0.067)
Il1b			. (0.051)
Tlr2			*
Tlr4	. (0.091)	**	**
Cpt1a	***	*	. (0.0539)
Ppara	*	***	
Elovl6	*		**
**FAs**			
Total FA		*	
C18 [% total FA]	***	*	
C16 [% total FA]	***	*	
C18:2 [% total FA]			
C18:1 [% total FA]	***	*	
C18:0 [% total FA]	***	**	
C16:0 [% total FA]	***	*	
C16:1 [% total FA]	***		
Ratio C18:C16	***	*	
